# Tracing down the updates on Ebola virus surges: An update on anti-ebola therapeutic strategies

**DOI:** 10.2478/jtim-2023-0100

**Published:** 2023-09-02

**Authors:** Shiza Malik, Yasir Waheed

**Affiliations:** Bridging Health Foundation, Rawalpindi, Punjab 46000, Pakistan; Office of Research, Innovation, and Commercialization (ORIC), Shaheed Zulfiqar Ali Bhutto Medical University (SZABMU), Islamabad 44000, Pakistan; Gilbert and Rose-Marie Chagoury School of Medicine, Lebanese American University, Byblos 1401, Lebanon

**Keywords:** ebola virus, ebola virus disease, vaccines, anti-viral agents, drugs, therapeutic approaches, novel drugs

## Abstract

Ebola virus (EBOV) related health complications have presented a great threat to the healthcare system in the endemic regions. The outbreaks of 2013-2016 and 2018-2020 brought along a huge healthcare burden for the afected communities. Knowing the seriousness of the matter, a series of research experiments have been actively carried out to devise efective therapeutics, drugs, and vaccination protocols against the Ebola virus disease (EVD) in the past decade. The purpose of this piece of literature is to shed light on vaccination protocols being clinically evaluated for EVD. A methodological approach has been adopted to gather relevant data from the latest publications. The compiled data include the molecular mechanistic insights into Ebola infection and a brief overview of diferent vaccination strategies: inactivated and DNA vaccines, virus-like particles and replicons, reverse genetic and recombinant approaches, entry, ion, and gene expression inhibitors, and some repurposed drugs. This data will help the scientific community to get a comprehensive overview of therapeutic interventions against Ebola that could be related to modifying EBOV vaccines and designing other antiviral vaccinations. Having said that, further work in modern therapeutic design is pertinent to tackle and lessen the healthcare burden expected from such outbreaks in the future.

## Introduction

Ebola viruses (EBOV), are members of the Filoviridae family of viruses; they have biology consisting of a negative-stranded RNA structure. Besides some other disease-causing filoviruses, EBOV has gained global attention for its links with Ebola virus disease (EVD) outbreaks, which have been observed over some past decades. These are of zoonotic origin and African fruit bats are considered the natural reservoirs of EBOV. From these birds, the viruses get transferred to larger mammals and from there to humans. Over the years EBOV has largely transmitted to the human population mainly through routes of body fluid contact and transmission.^[1]^

EVD, is the major manifestation of EBOV in humans. It is characterized by its lethal transmissible feature that involves high fatality rates, severe fever, arthralgia, and hemorrhagic symptoms which sometimes lead to sudden shock, organ failure, or death.^[2]^ Epigenetic factors including young age, sex, and pregnancy, are considered as deleterious prognostic factors that may lead to EVD development and viral proliferation.^[3]^ Sometimes patients develop other disease symptoms surrounding immune system dysfunctionalities such as auto-immune dysfunction, rheumatoid arthritis (RA), systemic lupus erythematosus (SLE) or spondyloarthritis and uveitis etc.^[4]^

EBOV remains in infected bodies for a longer period and prolongs disease transmissibility. Dysregulated immune responses help further viral spread to vital organs where EBOV works to cause multiple organ functional failure. Sometimes before the severe form of organ dysfunction develops, the patient’s immune system responds by developing a robust immune defense which leads to the clearing of viremia load in the resolution phase. Otherwise, a long-lasting viremia loading in the body leads to large-scale disease spread and transmission which ultimately drives patients to severe health conditions and sometimes to mortality states. Though EVD has mostly remained endemic to some regions, its cases are often reported in regions far from endemic countries and this creates a threat of further viral spread in the form of an epidemic.^[1]^

The recent outbreak in Africa from 2013-2016 was an alarm for scientists to be more active against EBOV mitigation measures. Therefore, various research studies have been conducted in the past to understand the molecular biology and viral molecular biology, infectious life cycle and underlying immune system response towards viral infection in animal and human models. All these studies were necessary to develop insights into the viral infection, host interaction, and disease development mechanism. The sole purpose has been to develop effective management, preventive and therapeutic strategies against such disease-causing agents.^[5]^

With the help of EBOV-related studies, excessive knowledge and understanding have been developed for EBOV and EVD, and now scientists are trying to manufacture upgraded antivirals and vaccines to control the endemic in its acute form. In this review we will try to bring updates regarding the latest understanding of Ebola molecular biology, life cycle and host interaction; some overviews about the nature and past incidence of Ebola virus outbreaks will also be discussed. Moreover, the major focus of this study will be kept on the recent antivirals and vaccine-based therapeutic strategies that are coming to the surface against EBOV.

## Methodology

This review article has been compiled via a methodological approach to include only the most recent and most up-to-date studies. The search was limited to the period of 2015-2022. Different search engines that were thoroughly searched for compiling this piece of literature include: Google Scholar, PubMed, NIH (National Library of Medicine), and Web of Science. Additionally, statistical readings have been adopted from the official websites of World Health Organization (WHO), Food and Drug Administration (FDA) and Center for Disease Development and Control (CDC). The major search items have been the Ebola virus, Ebola endemic, Ebola outbreak, Ebola virus disease, vaccines against EBOV, and antiviral drugs against EBOV, among other related terms. Moreover, studies of only English language origin have been made part of this literature. This study is not sponsored by any funding and does not involve any clinical study that may require ethical approval or informed consent.

## Results

### Ebola virus - Molecular Biology

Some insights that have been developed up till now related to EBOV will be discussed briefly here. EBOV is an enveloped, negatively-stranded RNA virus with an approximate diameter of 80 nm and a length of some hundreds of micrometers. The viral genome has been studied and has seven major structural proteins namely, the nucleoprotein (N), and the virion proteins (VP): VP24, VP35, VP30, VP40, the glycoprotein (GP), and RNA polymerase (L). Nucleoproteins enclose the viral RNA in the form of a ribonucleoprotein complex and bind further to other structural proteins including VP35, VP30, and L.^[6]^ Like other major infectious viruses, EBOV takes entry into the host cell with the help of its membrane GP. The ribonucleoprotein complex interacts with this envelope through the help of matrix proteins VP 40 and VP24. EBOV infection is not limited to a certain cell type; a variety of host cells allow viral interaction and entry, such as fibroblasts, hepatocytes, and adrenal cortical cells. Moreover, a variety of immune system cells are also involved in viral entry and life cycle and these may include monocytes, macrophages, and dendritic cells among others.^[3]^

Following entry into the host, the viral genome is engulfed by a process of non-selective internalization called macropinocytosis. This internalization is supported by other cellular entities and attachment factors of the host cell such as C-type lectins, T-cell immunoglobulin, mucin domain 1, sphingomyelinase (aSMase), plasma membrane and tyrosine kinase receptor Axl.^[7]^ The major viral entities that play a vital role in this process include surface GP as elaborated earlier, and class 1 fusion proteins. GP, through its receptor binding subunit (GP1) and fusion conducting subunit (GP2), carries out a successful fusion process. Chemical linkages like disulfide and covalent bonding are involved in host-viral interactions. After successful host entry, the virus moves further into the endolysosomal pathway leading to late endosomal formation.^[6]^ Here cysteine proteases cathepsins B and L play an important role in processing GP1 protein to convert it into a fusogenic form. These cathepsins are low-pH-dependent proteins. As a result of the fusogenic form, the putative receptor binding domain is exposed and it allows GP1 and endosomal proteins Neimann-Pick C1 (NPC-1) interaction. The resultant outcome is the G2-dependent fusion of viral envelope with endosomal limiting membrane.^[7]^

**Figure 1:**
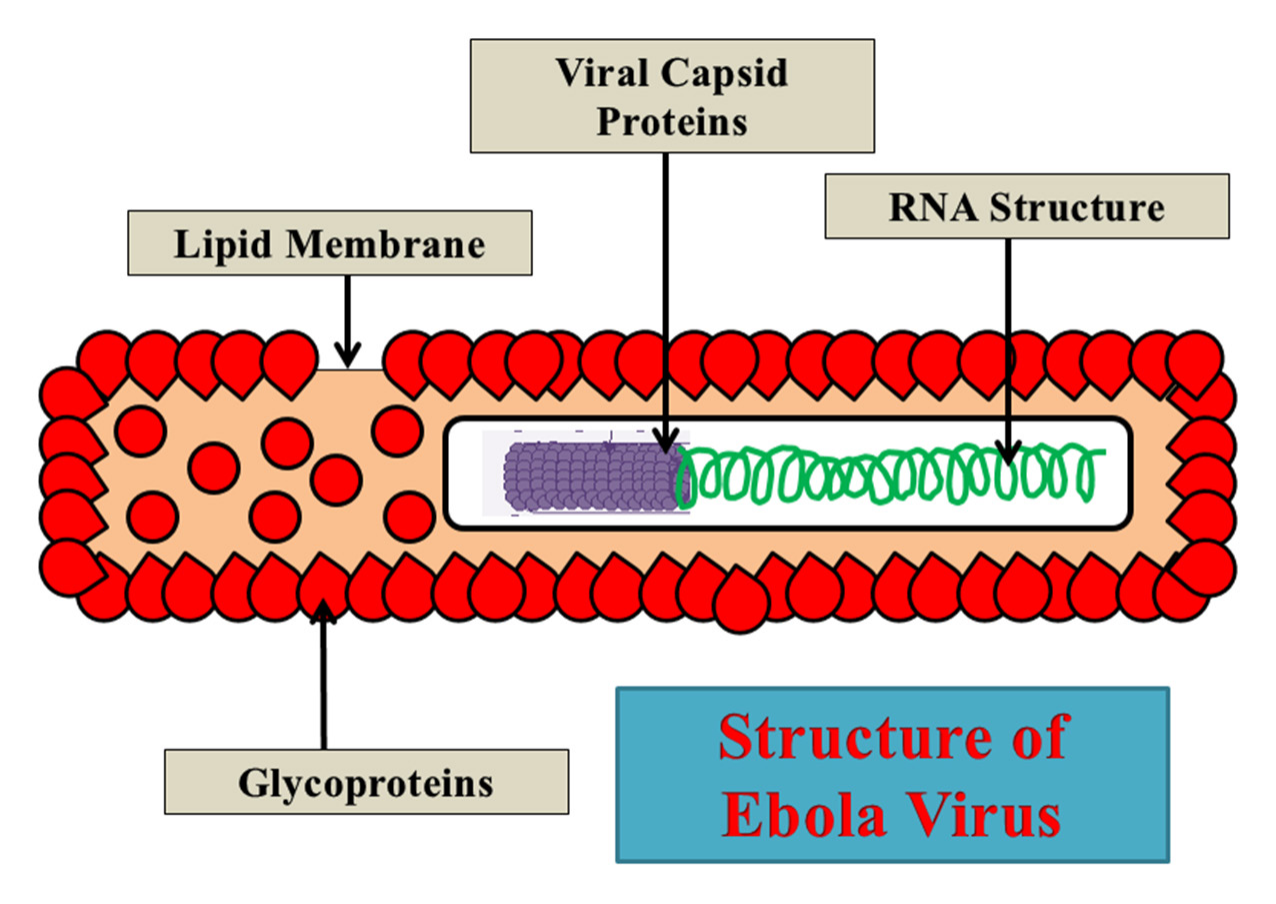
Ebola virus structural representation.

Some studies have conducted a further inquiry on the fusion mechanism and have found that fusion and entry steps also require the help of Two-Pore Channel 2 (TPC2) activity, although further data is required to validate its role. As a result of all these interconnected events, the viral genome is entered into the host cell cytoplasm which further undergoes transcription and replication protocols to multiply its genome. Finally, after processing, assembling, and budding of new viral genes, the cycle of neighboring viral-cell interaction starts. The detailed analyses of these stages are beyond the scope of the current study; however, comprehensive data is available regarding the viral replication process within the host cell. ^[6,8]^ These studies help scientists to understand host-viral interactive sessions and how antiviral strategies could be proposed to hurdle and block any of the activity in this life cycle. A diagrammatic representation of the simplest Ebola virus structure is shown in Figure 1.

### EBOV - Epidemiology and Outbreaks

The origins of EBOV outbreaks and diseases have mostly remained unknown, though scientists have proposed their zoonotic origin from endemic regions. EBOV has been reported to be transmitted directly through human-to-human contact with body fluids or contaminated fomites.^[5]^ The zoonotic inference could be deduced from the natural host reservoirs of EBOV - fruit bats. Moreover, the inference related to fluidic transmission in human-human interaction has been driven by the observational analyses of viral loads into breast milk, saliva, urine, cerebrospinal fluid, aqueous humor, amniotic fluid, tears, skin swabs, stools, and blood derivatives.^[1]^

The first outbreak was reported in 1976 in the Democratic Republic of the Congo (DRC); since then, more than 17 outbreaks has been reported in African countries. The most recent outbreak was observed in the period 20132016 within western African countries. As the outbreaks are ongoing, their endemic nature is slowly reverberating into an epidemic nature. Moreover, the data regarding outbreaks are limited, thus creating uncertainty regarding the regional disease spread range in the world. ^[7, 8, 9]^ Statistical data regarding the occurrences of EVD in specific endemic regions and specific years can be traced from the official website of CDC.^[8]^ All the studies that have been conducted to track epidemiological outbreaks of Ebola show that Ebola accounts for 50% chances of death in patients who develop EVD.^[9]^ EBOV Resurgence and mitigation needs.

To tackle EVD spread, certain mitigation and adaptation protocols for disease management have been introduced by scientific and healthcare communities. These measures may include measures about governance, communication, isolation, infrastructural development, and international travel and connectivity. Moreover, more steps are being taken to outline the surveillance of disease.^[10]^ The very word outbreak is used when an endemic adopts the form of an epidemic by reporting more cases than the range of an endemic. Typical exposure to Ebola in lower-income countries and rural locations often delays its outbreak reports to other regions. Moreover, the limited facilities in these regions further curtail the process of awareness and mitigation measures in affected regions, mainly the middle African regions (the DRC, Gabon, and the Republic of the Congo). These regions were prime areas of EVD spread but slowly the scale of viral infection got upgraded especially during the outbreak of 2013-2016.^[11]^ This sudden outbreak broke the assumption of the endemic nature of this exotic pathogen and presented another challenge for public health globally. This case was taken into notice by higher healthcare authorities and soon WHO declared Ebola to be of wider scale influence rather than just an endemic.^[9]^ This measure has transformed the nature of EBOV and has raised concern among scientific communities to bring the latest antiviral therapeutics against EVD.

A more recent outbreak in DRC (2020), Guinea (2021), and Uganda (2022) has shown large-scale destruction in the form of infected cases and resulting mortalities.^[9]^ Furthermore, these viral resurgences have suggested a new paradigm shift in outbreaks which has rung alarm bells for the healthcare community to devise a long-term vaccination and socio-environment strategy against EVD and other viral infections.^[12]^ The surviving patients of these outbreaks were a motivating factor for the scientist to design appropriate vaccination protocols against viral pathogenesis. The outbreak’s epidemic and widespread nature coupled with the rapid pace of scientific interest and research outcomes makes it imperative to employ the latest therapeutics in clinical trials and get official licensure for marketing so that the Ebola-induced healthcare burden could be effectively dealt with. Any more delay in real-time application of therapeutic applications would be ineffective against the latent resurgence of EBOV infection.^[13]^

### Anti-EBOV Drug regimens and supportive medication

Since the first outbreak, scientists have been working to formulate effective therapeutic strategies against EVD. Some therapeutic interventions have been taken to clinical trial phases. Currently, several vaccines are in research annals. Moreover, one of the vaccines has also been approved by FDA, namely the rVSV-vectored vaccine, which has been shown to provide nearly 100% protection against EBOV in some cases.^[14]^ An important reason for active research trials of such therapies is to curtail any such outbreaks of endemic, epidemic, and pandemic nature that have already put immense pressure on the healthcare system in the preceding years.

The threats of Ebola outbreaks have pushed scientists to design antiviral regimens of various kinds. This success is important because working with Ebola viruses is highly dangerous and only a few labs in the world with biosecurity level-4 containment checks can handle and work on the Ebola virus. Therefore, approved vaccination and antiviral options are a big success from the scientific community. In the coming sections, we will shed some light on the approaches on which they have been working to formulate EBOV vaccination candidates.^[15]^

Some supportive medical options have also been introduced during this period of Ebola outbreaks. These include the application of immune modulatory substances such as broad-spectrum antibiotics, including monoclonal antibody MAb114, remdesivir, favipiravir, biological products such as REGN-EB3 and ZMapp, opiates or anti-emetic medications, and non-steroidal anti-inflammatory drugs.^[16]^ These therapeutics have been designed mostly by reverse genetics and in-silico approaches. They mostly worked by inhibiting or blocking the function of viral genes or pathways through which viruses divide and cause infection in the body.

Additionally, the use of disease-preventing medications also included oral crystalloid fluids and anti-coagulants that proved to be useful for maintaining electrolyte balance in patients’ bodies. In extremely ill patients the use of ventilation, surgeries, and renal replacement therapies, have also shown confinement against mortality.^[17]^ The basic strategy involves preventive and treatment options that may be adopted at various stages. Preventive medication consists of the application of protective vaccines in members of vulnerable communities. While treatment options are projected for confirmed EVD patients and may include the previously discussed options such as therapeutic antivirals. These antivirals help to control disease symptoms, besides curtailing further viral loading and transmission.^[16]^

### Anti-EBOV-Vaccine development process

As explained earlier, the Ebola epidemic has burdened the healthcare community in the past decade and therefore vaccination drives are within research annals since then. Some viral vaccination development has moved forward in the form of replicative and non-replicative vector-based vaccines. In non-replicative viral vectors, some important genes are deleted that are crucial for viral replication such as GP. Such vaccines have high tolerability and must be introduced in higher dosages to give significant responses. On the other hand, the replicative vaccine uses replicative vectors that code for specific viral antigens for activating immune responses. These antivirals are injected in low doses and have lower tolerability.^[18]^

Some other strategies include the use of inactivated viruses, virus-like particles, DNA-based vaccines, mutant viruses, heat-inactivated viruses, and some new candidates being introduced to the market, such as those driven from plant sources and nanobiotechnology.^[19]^ The FDA-approved candidate vaccine belongs to the live-attenuated recombinant vesiculo viruses. Now scientists are trying to formulate combinatorial therapies to bring more useful future outcomes in disease management.^[20]^ Moreover, data is being compiled to check the real-time efficiency of vaccine candidates. For example, some vaccination drives have been conducted in DRC and as of 2020, approximately 280,000 individuals were vaccinated, of which approximately 94,000 reflected lower viral loads as compared to unvaccinated individuals.^[21]^ This result established A grade vaccination efficiency. Similarly, some other studies were also conducted to gather these statistical analyses. Based on these results, similar vaccination candidates are being used in DRC to curtail the danger associated with post-EBOV exposure prophylaxis, especially in healthcare workers. This is essential to globally establish an Ebola vaccination campaign for the proper eradication of this healthcare burden.^[3]^

### Vaccines, drugs, and antiviral agents against Ebola

**Figure 2:**
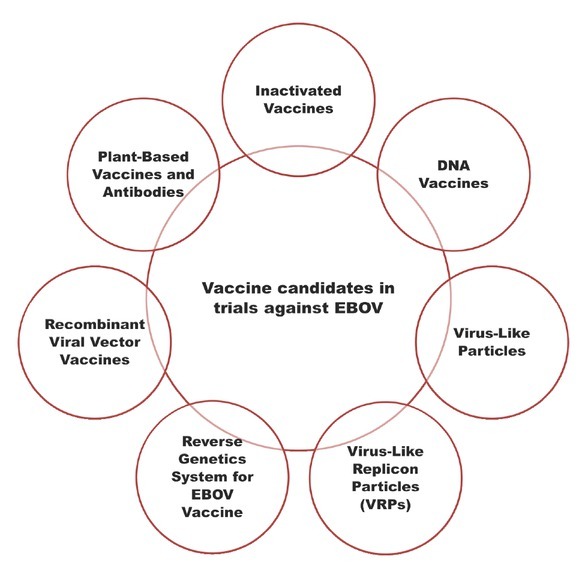
Diagrammatic summary of Different vaccine candidates in trials against EBOV. EBOV: Ebola virus.

**Figure 3:**
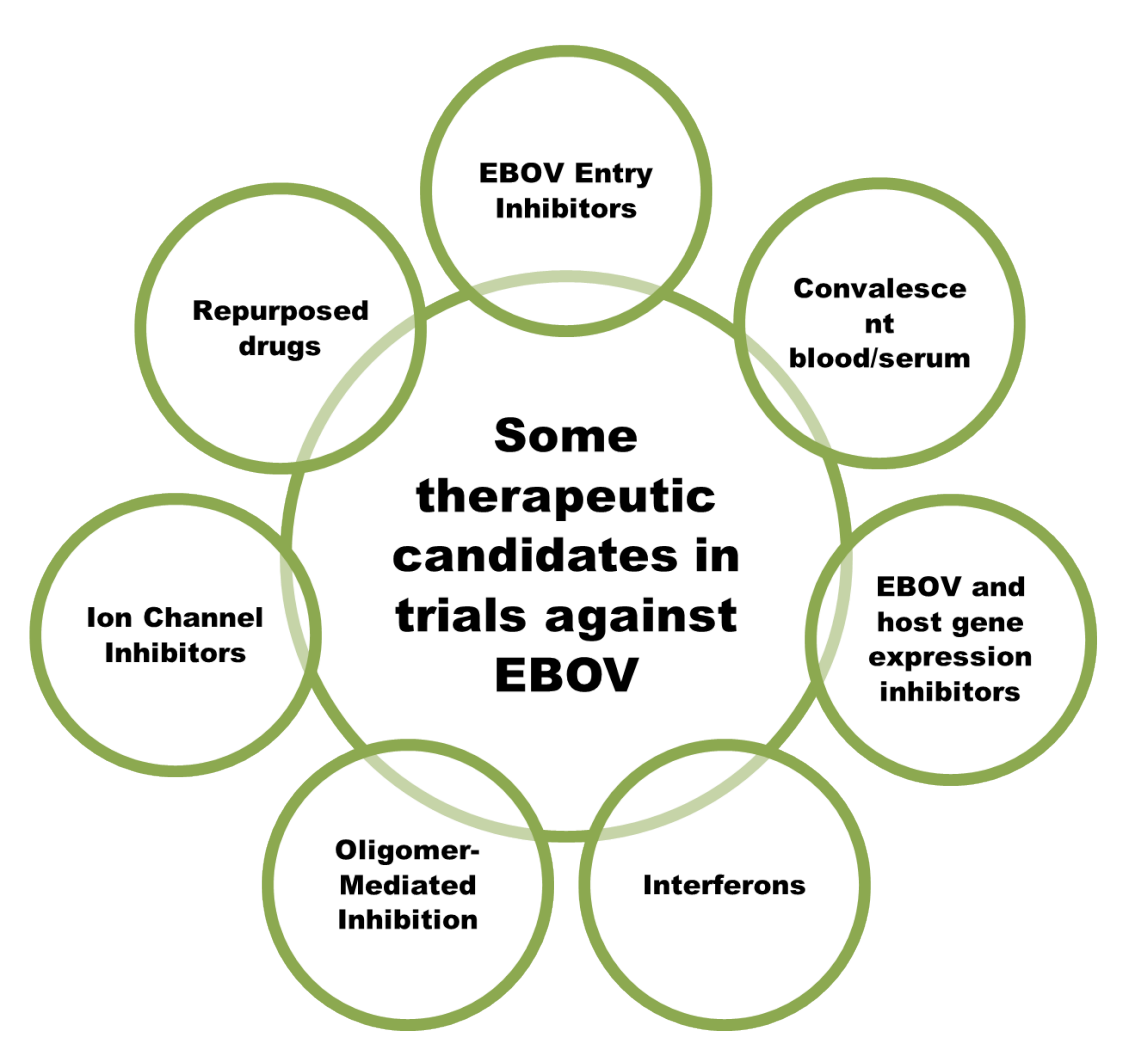
Diagrammatic summary of some other therapeutic candidates in trials against EBOV. EBOV: Ebola virus.

A brief overview of different vaccine candidates that are in various trial phases are shown in Table 1 and Figure 2. Alongside, some drugs and antiviral agents against EBOV has been included in Table 2 and Figure 3. It should be noted that comprehensive detail on each of these therapeutic candidates could be studied in the respective research associated with them, however, here we will only present a glimpse for readers to understand the progress linked with the Ebola therapeutic drive worldwide.

**Table 1:** Different vaccine candidates in trials against Ebola viruses

**Sr. no.**	**Type of vaccines**	**Brief overview of Mechanism of action**	**Related Vaccine candidates under trial**
1.	Inactivated Vaccines	Non-replicating viruses are used to design inactivated vaccine candidates. Trials are being conducted to reduce the reversion of virulence and increase potency, safety, and efficacy against EBOV infection. Heat, radiation, or chemical agents like formalin are used for inactivating viral agents.^[22]^	Liposomeencapsulated Irradiated EBOV
2.	DNA Vaccines	In this protocol plasmids are designed to express specific antigens that are capable of generating both innate and adaptive immune responses. It is a rather easy and cost- effective process. These vaccines are sometimes boosted with attenuated forms of viruses. Mostly they are in the experimental phase and have proven safety but they only create a short immunity development span which requires repeated vaccination dosages against. These developments indicate that further work is required to make DNA vaccines more efficacious for EBOV. ^[14,23]^	EBOV-GP-DNA. Combine DNA plasmid vaccines comprising GP, NP
3.	Virus-Like Particles	Virus-like particles are being progressively used to design modern vaccines for various infectious diseases. In the case of Ebola VLPs (EBOV-VLPs or eVLPs), the design includes the expression of transmembrane glycoproteins (GP) and structural proteins (VP40) within mammalian cells. These proteins impart characteristic morphological features like EBOV. Moreover, they elicit immune responses of both innate and adaptive origins quite similar to original viruses. Some modern vaccination approaches include manufacturing at nanoscales which increases the efficacy and thermostability of VLPS. Results indicate that by adjusting the vaccine dosages, effective vaccine candidates could be designed in the future based on VLPs. ^[16,24]^	Baculovirus-derived eVLPs Nano-VLPs
4.	Virus-Like Replicon Particles (VRPs)	As the name indicates viral replicons are similar to live-attenuated viruses. In their design, viral replication genes are kept but structural genes are deleted and replaced with immunogenic genes. Filoviruses and alphaviruses are mostly used for this design. These VRPs undergo replication and transfection without making the viral progeny in competent cells for up to one cycle since they lack structural genes. The proteins that are most commonly used are GP and VP24, VP30, VP35, and VP40. The benefit of using VRPs is the cancellation of viral pathogenic reversion and the generation of strong humoral and antibody-mediated responses. ^[22,25]^	Venezuelan equine encephalitis virus (VEEV) based VRPs. Alphavirus Semliki Forest virus-based recombinant replicon vector DREP VRP-EBOV GP VRP-SUDV GP
5.	Reverse Genetics System for EBOV Vaccine	In this design reverse genetics approach is used to prepare replication incompetent viral; particles that lack essential structural proteins. The manufactured particles are replication-deficient. The dose efficacy has proved to be very strong in trial experiments. Strong chemical like hydrogen peroxide is used to inactivate virus particle to overcome their likely recombinant potential in this protocol. However, these chemical does not affect the immunogenic character of designed vaccines. ^[15,26]^	EbolaΔVP30
6.	Recombinant Viral Vector Vaccines	This vaccination design is gaining popularity for linked induction of CMI responses. The antigens are expressed and processed within the cytoplasm and thus elicit strong immune responses. Virus species such as Replication-competent rVSV and chimpanzee adenovirus 3 (ChAd-3/cAd3) are progressively being employed for recombinant vaccine designs. The experiments have shown a lack of viral shedding and almost nonexistent disease symptoms in model organisms which pertains to their high efficacy and safety measures. ^[20,27]^	(rVSVΔG/EBOVGP) 977 ). rVSV-Zaire EBOV rVSV-EBOV vaccine GamEvac-Combi, EBOV GP (AdC7-GP) (cAd3-EBO) ChAd-3 vectored VSVΔG-HA-ZGP MVA-BN-Filo. HPIV3/EboGP.
7.	Plant-Based Vaccines and Antibodies	RNA and DNA viruses are being modified to serve in plant-based vectors system and express several heterologous proteins. Major EBOV antigens such as GP, NP, and VP gene family, all of which elicit strong immune responses, are used to design vaccines by manufacturing these proteins in different plants. Genomic transfer is carried out through viral vectors and expression develops in a transient manner. Plants serve as a bioreactor to generate bulk production of antibodies mainly through the process of agroinfiltration. Lettuce plant is a good plant host for such vaccines. This approach is rather old and but is commercially improved in designing viral proteins in order to meet the high manufacturing demand for Ebola therapeutics. For the large-scale commercialization of these designs, it is counted as one of the modern vaccinations designs that is increasingly being employed for epidemics of Influenza, EBOV and other pathogens. ^[22,28]^	ZMapp-*N. benthamiana*-derived antibodies. Phoolcharoen – tobacco plant-derived antibodies. EBOV VP40 – designed through tobacco plants. EBOV mAb13F6-magnICON expression system.

**Table 2:** Some other therapeutic candidates in trials against EBOV

**Sr. no.**	**Type of therapeutics**	**Brief overview of Mechanism of action**	**Candidates under trial**
1.	EBOV Entry Inhibitors	Endosomal proteases play an essential role in EBOV infection following its host cell entry. By designing antagonists to these enzymes, EBOV trafficking, maturation and infection can be averted. Moreover the clathrin-dependent endocytosis with evident roles of NPC1 receptor, GP proteins, TIM-1, TIM-4, and Axl (a receptor tyrosine kinase) for entry of enveloped EBOV provides essential information for designing entry inhibitors against EBOV. These proteins are targeted and blocked in different methods to design particular viral entry inhibitor drugs. ^[27,29,30]^	Silvestrol extracted from *Aglaia foveolata *Single siRNA candidates- benzylpiperazine adamantane diamide-derived compound-Tetrandrin. Clomiphene and toremifene (ER drugs) Amiodarone, (an ion channel blocker) Dendrimers and fullerene C^60 ^Clarithromycin and posaconazole, (anti-fungal compounds) *5-(N-ethyl-N-isopropyl)* amiloride MLS000394177 and MLS000733230 *Prunella vulgaris*, a Chinese herb, (HIV) -1-based vector system. Quercetin 3-β-*O*-d-glucoside (Q3G), a flavonoid derivate. Synthetic serine protease inhibitor, nafamostat mesilate (NM) -CatB inhibitors). Chemically modified human serum albumin with 3-hydroxyphthalic anhydride (HP-HSA)
2.	Transfusion of Convalescent blood/serum- emergency responses.	Since the Convalescent serum of infected individuals contains immunoglobulins against EBOV and lacks red blood cells and platelets, therefore the blood of infected and /or vaccinated individuals could prove to be beneficial for vaccination. This approach has been approved by WHO for emergency responses against critical EBD. ^[9]^ Though efficacious this method requires intense prior screening to avoid infection with any left EBOV active residues, or other pathogenic viruses including HIV, HPV, HBV, and HCV in donor’s blood. Additionally, sometimes passive immunization is done with the transfer of neutralizing antibodies (driven from infected persons) to individuals suffering or recovering from EVD. This treatment option has shown efficacy in a number of case studies. mABs basically neutralize the entry mechanism of viruses and hence avert infection. These antibodies are presenting very impressive results in mouse models hence their future vaccine applications are totally in scope. However, epitope mutations and the need for high dosages are the problems that need to be dealt with for better applications of this therapeutic protocol^. [15,29,31^]	mAb114 and MB-003, ZMAb, ZMapp, and MIL-77E mABs cocktails (KL-2E5 and KL-2H7) mABs FVM04 (a mAb) Fab, KZ52 Bispecific Trojan-horse antibodies Cell-penetrable human scFvs (HuscFvs) (transbodies) The cell-penetrable small antibody fragments (HuscFvs) or superantibodies
3.	EBOV and host gene expression inhibitors	Just like viral entry inhibitors, viral genomic expression inhibitors are also being designed for vaccination protocols. These candidates work by inhibiting a specific gene either in the host cell or in viral genetic material for hurdling the replication and infection cycle of the virus. They may inhibit certain regulatory signaling networks within the host cell such as MAP-Kinase. However, more work is required in this domain to design effective therapeutic designs. ^[15,18,32,33]^	Cationic porphyrin TmPyP4 BCX4430 (a nucleoside analog) -viral RNA polymerase inhibitor The eukaryotic initiation factor 5A (eIF5A) blockers Hypusination and spermidine blocker *Selective Estrogen Receptor Modulators: *toremifene and clomiphene *Protein Kinase Inhibitors: *(Sunitinib and erlotinib), apilimod pyridinyl imidazole Isothiazolo [4, 3-b] pyridine-based inhibitor of GAK (Cyclin G Associated Kinase) 1-Benzyl-3-cetyl-2-methylimidazolium iodide benzodiazepine (“compound 7” or MBX2254 and MBX2270) Iminodyn 17 -an inhibitor of the GTPase activity. Retro-2 Diaryl-quinoline compounds Pep-3.3 U18 666A
4.	Interferons	Interferons show a potent inhibitory effect against EBOV. For this reason, they are often used for the treatment of EVD. They effectively conduct viral clearance from the blood stream and limit further disease symptoms. However, their application is coupled with other side effects in form of fever, myalgia and others. ^[30,34]^	Tilorone hydrochloride
5.	Oligomer- Mediated Inhibition	Antisense therapies employing small interfering (si) RNA have shown good targeting abilities against different genes of EBOV such as the RNA polymerase L protein. These siRNAs are sometimes used to block micro RNAs that are essential for viral infection mechanism, hence the compound working mechanism of miRNA and siRNA needs to be properly formulated to prove their immunomodulatory responses in patients. ^[17,32,33]^	AVI 6002, AVI-6003, AVI-7537, TKM-130803 LNPs/siRNA (TKM-Ebola), The inhibitors of hsa-miR-1246, hsa-miR-320a, and hsa-miR-196b-5p A sulfonamide (MBX2254) and a triazole thioether (MBX2270)
6.	Ion Channel Inhibitors	Filovorus including EBOV mainly enter host cells through various ion channels. Following this observation, some drugs have been proposed by scientists that have been tested to block ion channels and thus inhibit virus entry into the host cell. However, these ion inhibitor drugs are not solely applied yet coupled with other viral vectors such as EBOV or Marburg virus (MARV) glycoproteins. Further work is needed to prove efficacy of drugs based on this therapeutic dimension. ^[35,36]^	amiodarone, dronedarone, verapamil, methyldiethanolamine amiodarone, dronedarone, verapamil, methyldiethanolamine Bepridil- and tetrandrine drived - calcium channel blockers Noricumazole- potassium channel inhibitor
7.	Repurposed Drugs	Some old drugs manufactured previously for other purposes are being checked against EVD to evaluate their possible effect on disease manifestations. This is essential until a fully efficacious drug or vaccine is available in the market against EVD. These drugs work by various mechanisms such as viral entry inhibition, replication inhibition or by eliciting immune responses. Moreover, combine therapeutic approaches are also being adopted for these drugs in order to increase their efficacy and reduce the side effects associated with their application.^[34,37]^ These may include several categories of drugs such as Antiparasitic Drugs, Antibiotics Drugs, antifungal drugs, Psychoactive Drugs and Antidepressant drugs among others.^[1,16,22,23,27,28,36,38]^	Amiodarone Dronedarone Verapamil Statins Angiotensin-converting enzyme inhibitors Angiotensin receptor blockers Phosphoinositide 3-kinases inhibitor LY294002 Calcium/calmodulin kinase (CAMK2) inhibitor KN-93 p38 mitogen-activated protein kinase inhibitor SB202190 Estrogen re-uptake modulators (toremiphene and clomiphene) Brincidofovir, a cidofovir analogue Alisporivir Emetine–cephaeline-analog Rosuvastatin, atorvastatin, and pravastatin Anti-malarial drugs such as: chloroquine, hydroxychloroquine, pamaquine, primaquine, and plasmaquine. Esomeprazole and omeprazole. the anti-trypanosomal agent, Suramin (Germanin or Bayer-205). Favipiravir and the pyrazine carboxamide derivative T-705 anticancer agents (vinblastine, vinorelbine/navelbine, and vincristine) Colchicine Indinavir, an HIV protease inhibitor Maraviroc, Abacavir, Telbivudine, and Cidofovir Enfuvirtide Vancomycin Bleomycin Octreotide Lanreotide, Somatostatin, and Ubidecarenone (CoQ10) . Nucleotide Analog Prodrug- GS-5734 Favipiravir (T-705), Ribavirin Lamivudine, Gossypetin and Taxifolin Adenosine nucleoside analogs such as BCX4430 GS-5734 *Antiparasitic Drugs: *Chloroquine, Amodiaquine, Hydroxychloroquine, Aminoquinoline, Artesunate-amodiaquine, Ferroquine, Suramin, Emetine, Albendazole and mebendazole *Antibiotics Drugs: *Teicoplanin, azithromycin *Antifungal drugs: *Terconazole and triparanol *Psychoactive Drugs: *Chlorpromazine imipramine *Antidepressant drugs: *Sertraline, Maprotiline, Trimipramine, Promethazine, Diphenhylpyraline, Ketotifen, Diphenhydramine and chlorcyclizine

### Approved EBOV vaccine and future implications for therapeutic interventions

Many factors restricted licensure of the EBOV vaccine, including its endemic nature of outbreak, slow research work, limited research labs, limited EBOV-related data, and financial hurdles for vaccine licensing.^[29]^ However, in the recent years of EBOV outbreaks (2015-2022), some improvement and speed have been acquired in vaccination processes. Now many vaccines have been given approval by FDA for level III laboratory experiments.^[36]^ Most of the approved vaccine candidates come along with improved efficiency, reduced side effects, low dosage needs, and cost-effectiveness.^[33]^ Ervebo (rVSV-EBOV; V920) is the only FDA-approved drug for human application yet other drugs in this list are also being tested on human subjects, for establishing their efficiency. This may include rAd5-EBOV, rVSV-EBOV (also known as rVSVΔG-EBOV GP or rVSV-ZEBOV), rChAd3-EBOV prime with or without MVA-BN-Filo boost, rAd26-EBOV prime with MVA-BN-Filo boost, rAd5-EBOV, Zabdeno/Mvabea (Ad26-ZEBOV/MVA-BN-Filo). ^[16,19,38]^

**Figure 4:**
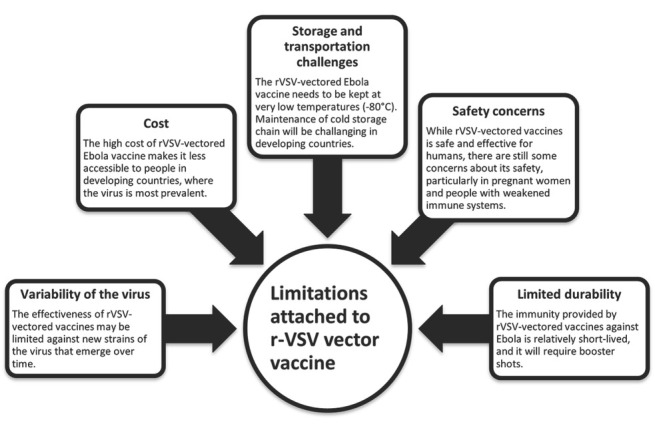
Limitations attached to r-VSV vector approved vaccine against EBOV. EBOV: Ebola virus.

For now, rVSV-EBOV vaccine has shown good results in level II and III clinical trials and thus has been projected for real-time application in patients as level IV trials. Until now it has shown good clinical outcomes in terms of safety, immunogenicity, limited side effects, and high efficacy in humans.^[29]^ For this reason, it is currently being employed in African endemic regions. The results of these experiments show that rVSV-EBOV has served its prime purpose of vaccination against EVD.^[38]^ It should be noted here that although the rVSV-vectored vaccine has shown nearly 100% protection against EBOV in some cases, it still has some limitations, as elaborated in Figure 4.

## Conclusion

The development process of Ebola vaccination has continued since 1980, and to date, only a minute number of drugs and vaccines have gone through upgraded clinical trials. Currently, research work is focused on investigating underlying molecular interactions and cellular entities involved in EVD. Bioinformatics approaches are progressively being employed to generate rapid outlines of vaccination design. These in-silico experiments are an essential and rapid alternative to the traditional vaccination design approaches. Keeping this observation in mind, it is time to redefine the vaccination strategies towards modern approaches, including nanomedicine and organic pharmaceutical as they are showing promising results for other viral diseases. The redesigning strategy is necessary to overcome the side effects, increase the vaccination design pace and make them more cost effective and efficacious at the molecular level. Moreover, the clinically approved drugs still need to be investigated until proper statistical results support their efficacy. Besides treatment options, appropriate mitigation, prevention, and adaptive measures need to be acquired and practiced by the healthcare community, the general public, and authorities. Moreover, community supportive care, healthcare measures, awareness campaigns, and participatory approaches from the public are of utmost need to prepare people for such outbreaks in the future.
